# Estimating Population‐Based Need for Lifestyle Interventions Among Young Adults With Mental Disorders in Australia

**DOI:** 10.1111/inm.70034

**Published:** 2025-04-04

**Authors:** Kate Gossip, Julie John, Charlotte Comben, Imogen Page, Holly E. Erskine, James G. Scott, Sandra Diminic

**Affiliations:** ^1^ School of Public Health The University of Queensland Brisbane Australia; ^2^ Queensland Centre for Mental Health Research Brisbane Australia; ^3^ Institute for Health Metrics and Evaluation University of Washington Seattle Washington USA; ^4^ Child Health Research Centre The University of Queensland Brisbane Australia; ^5^ Child and Youth Mental Health Service Children's Health Queensland Brisbane Australia

**Keywords:** Delphi, lifestyle, mental health, service planning, young adults

## Abstract

Lifestyle interventions encompass educational and treatment components addressing health risk behaviours such as sedentary lifestyles, nutrition, tobacco use, alcohol and drug use, sleep hygiene, and sexual health behaviours, and are known to improve symptoms of mental illness. However, these interventions are not widely available to young adults. The aims of this study were to (1) determine the proportion of young adults with mental illness who would benefit from lifestyle interventions and (2) describe the benefits and operational factors that should be considered when planning lifestyle interventions for young adults. A two‐stage approach was utilised, including structured consultation with experts in youth mental health (*n* = 12) and an online Delphi study with respondents with expertise and interest in lifestyle interventions (*n* = 14). The recommended proportion of young adults benefiting from lifestyle interventions varied between the structured consultation and Delphi study. Generally, the proportion increased with illness severity. Overall, study participants recommended that more young adults should have access to individually delivered interventions compared to group interventions. This study provides provisional estimates and operational details that could be used to increase the availability of lifestyle interventions for young adults, improving mental health, functioning, and physical health, and supporting improved life outcomes.

## 1 Introduction

Mental disorders are the leading cause of non‐fatal disease burden among young adults (< 25 years) worldwide (IHME 2019). Clinical symptoms of mental disorders, including low mood, anxiety, and cognitive impairment, can negatively impact social and occupational functioning. For example, young adults (18–24 years) with mental disorders may withdraw and become socially isolated from family and friends or develop sedentary lifestyles, leading to further mental and physical health complications.

Functional impairment associated with mental disorders impacts young adults at the time of illness and contributes to future adverse outcomes (e.g., through lower educational attainment and employment rates) (Curry et al. 2011; Di Rezze et al. 2016; Gibb et al. 2010; Knapp et al. 2016; Patel et al. 2007). Services that alleviate clinical symptoms, improve functioning, and prevent chronicity and disability are important for young adults (Colizzi et al. 2020; Cox et al. 2012; Jones 2013; Vos et al. 2015). However, up to two thirds of young people (12–25 years) accessing youth mental health services in Australia do not experience meaningful improvement in social or occupational functioning (Iorfino et al. 2022, 2018).

Lifestyle interventions are multidisciplinary interventions that seek to improve symptoms of mental illness, increase social functioning, and address risk factors for physical health comorbidity. Lifestyle interventions encompass educational and treatment components addressing health risk behaviours such as sedentary lifestyles, nutrition, tobacco use, alcohol and drug use, sleep hygiene, and sexual health behaviours. Recently, the evidence base for lifestyle interventions as a treatment for young people experiencing mental disorders has increased (Carney et al. 2021). Studies have shown benefits such as improved mood, motivation, sleep, energy, general functioning, and self‐esteem (Carter et al. 2016; Curtis et al. 2016; Watkins et al. 2020). Many interventions with positive outcomes have been nurse‐led or included nurses as integral members of multidisciplinary teams (Çelik İnce and Partlak Günüşen 2021; Curtis et al. 2016; Fernández Guijarro et al. 2019; Happell et al. 2023). Mental health nurses in particular are well placed to assess and respond to poor physical health, as well as promote and lead interventions to encourage healthy eating and physical activity or health behaviour modification for people with severe mental illness (Fernández Guijarro et al. 2019; Happell et al. 2019).

Evidence has led to the development of clinical guidelines and policy and practice advice that acknowledges the role of physical healthcare and lifestyle interventions in the treatment of mental disorders (Australian Government Productivity Commission 2020; Malhi et al. 2015; Manager 2019; Stubbs et al. 2018). However, lifestyle interventions are not a standard offering across mental health services worldwide (Bailey et al. 2019; Bartlem et al. 2015; Carney et al. 2021). Practical strategies have been suggested for addressing systematic barriers that impede the implementation of physical activity interventions into routine mental health care (Lederman et al. 2017). However, mental health service planners and clinicians require guidance on how many people are likely to need these interventions to support evidence‐informed resource allocation and service provision.

The Australian National Mental Health Service Planning Framework (NMHSPF) is a population‐based planning tool that estimates the proportion of the Australian population who are likely to need mental health services. The NMHSPF draws on a mixed‐method approach to quantify how many people need access to different types of mental health services and translates this information into service benchmarks (Diminic et al. 2022). A range of sources is used to enumerate service need at a population level including service utilisation data, peer‐reviewed literature, and survey data. Expert consensus is also used in the absence of evidence and data.

The NMHSPF has historically produced service benchmarks for infants, children, and adolescents (0–4, 5–11, 12–17 years), adults (18–64 years) and older adults (65+ years). However, there was no delineation of service benchmarks for young adults and no benchmarks relating to lifestyle interventions. Given a greater focus in Australia on delivering youth‐focussed mental health services (e.g., 12–24 years) and the growing body of evidence for lifestyle interventions, further research was undertaken to enhance the NMHSPF.

This study aimed to provide information and evidence to improve the planning and availability of lifestyle interventions for young adults in Australia. This was achieved by:

1. Defining the scope of lifestyle interventions and identifying the benefits and operational factors that should be considered when planning lifestyle interventions for young adults.
2. Generating expert consensus on the proportions of young adults with different levels of need who may benefit from lifestyle interventions in Australia.

## 2 Methods

**FIGURE 1:**
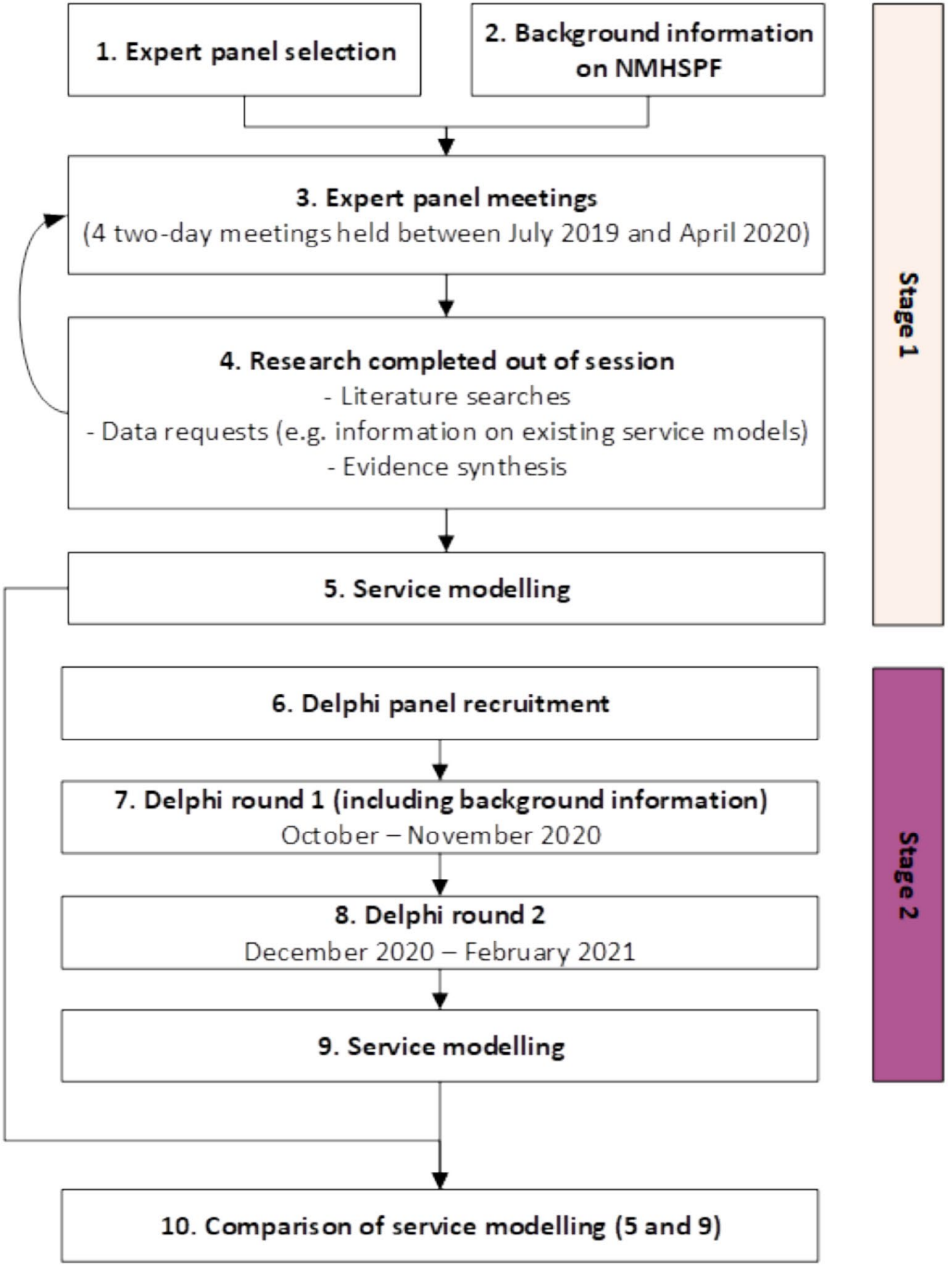
Two‐stage approach used in this study.

A two‐stage mixed methods approach was undertaken to determine the proportions of young adults (18–24 years) who may benefit from lifestyle interventions, including structured discussions with a group of experts in youth mental health and application of the Delphi method (Figure 1). This study was conducted as part of a broader piece of work to enhance the NMHSPF and was approved by the Human Research Ethics Committee of The University of Queensland, with Delphi participants providing implied consent through submission of survey Round 1 (Ethics ID: 2020001887) and a waiver approved for secondary analysis of aggregated Panel discussion notes (Ethics ID: 2020001878). Reporting of data and findings adheres to the Good Reporting of A Mixed Methods Study guidance (O'Cathain et al. 2017).

### 2.1 National Mental Health Service Planning Framework

Proportions requiring lifestyle interventions were estimated for young adults with sub‐threshold mental illness and diagnosed mental disorders as per the NMHSPF need groups (i.e., groups of people experiencing similar levels of impairment and complexity associated with mental illness). Each need group has an associated care profile. Care profiles estimate the proportion of people in the need group who may benefit from different service types, as well as the frequency and duration of each service required to provide good mental health care for the group (Diminic et al. 2022). Further NMHSPF definitions are provided in Data S1.

### 2.2 Stage 1—Face‐To‐Face Meetings With Experts (Steps 1–5 in Figure 1)

#### 2.2.1 Panel Selection

A youth mental health expert panel (henceforth referred to as the Panel) was formed in 2019 to support the development of care profiles for a new young adult (18–24 years) age group within the NMHSPF (Diminic et al. 2022; Gossip et al. 2022). Panel members were invited to participate via email and identified from a range of sources, including lived experience organisations, recommendations from the research team's professional networks and participants of past NMHSPF expert panels. Panel members had at least 5 years' experience working in youth mental health services or direct lived experience and collectively had experience working across all sectors of the Australian mental health system, including primary care, specialist public sector, and non‐government organisations. The Panel (*n* = 12) included seven youth mental health service managers, most of whom were also clinicians, four young adults with lived experience of mental illness (one of whom was also a carer of a young adult with mental illness), and one carer who was a parent of a young adult with mental illness (Data S2).

#### 2.2.2 Panel Meetings

The research team met with the Panel for four two‐day meetings between July 2019 and April 2020. During meetings, Panel members were asked to consider published evidence and draw on their practice‐based or lived experience to discuss and agree on the proportion of people in each need group who would benefit from and be likely to accept different types of mental health services, the average number and duration of occasions of service needed, and the workforce types best qualified to deliver services.

At times, there were differing views on what services should be included. Through iterative group discussions, Panel members were able to arrive at a consensus as to whether some young adults in a care profile would need access to a particular service. Minutes, including key decision points and action items (but not individual opinions or responses) were recorded by the research team and circulated to all Panel members for feedback.

Minutes from Panel meetings were then reviewed by the research team and discussion points relating to lifestyle interventions extracted. Discussion points were reviewed by two authors and grouped into three topics: purpose and scope, benefits, and operational factors. Young adult care profiles were also reviewed and information about the proportion of each need group who would benefit from lifestyle interventions as modelled by the Panel was extracted.

### 2.3 Stage 2—Delphi Method (Steps 6–9 in Figure 1)

Following Stage 1, the Delphi method was used to generate broader consensus on the proportion of young adults in each need group likely to benefit from lifestyle interventions. The Delphi method involves convening an expert panel to anonymously complete multiple rounds of targeted questionnaires (usually online) to develop consensus on a particular topic (Jorm 2015). This anonymity allows respondents to freely present opinions without being influenced by dominant opinions that may strongly influence group discussions. It also allows for participation from a range of experts from across Australia without associated travel and meeting costs.

#### 2.3.1 Delphi Recruitment

Purposive and snowball sampling were used to identify a broad pool of potential participants across Australia. Email invitations were sent to individuals who had previously participated in NMHSPF expert panels during 2018–2020, lead authors on sentinel Australian lifestyle interventions publications, professional societies (such as the Royal Australian and New Zealand College of Psychiatry, Exercise and Sport Science Australia), consumer and carer peak bodies, mental health research organisations, and mental health service providers.

#### 2.3.2 Delphi Design

Questionnaires were developed and implemented using *Checkbox*. The Round 1 questionnaire aimed to obtain opinions on the proportion of people in NMHSPF need groups who would likely benefit from lifestyle interventions. Respondents were asked to estimate the proportion of each need group who they thought would benefit from individually delivered or group‐based lifestyle interventions using a sliding scale (from 0% to 100% at increments of 10) (see Data S1).

The Round 2 questionnaire aimed to achieve consensus on the median score of each quantitative question and the key qualitative themes arising from Round 1, using a five‐point Likert scale (‘strongly agree’, ‘agree’, ‘neither agree nor disagree’, ‘disagree’ or ‘strongly disagree’). In both rounds, respondents were able to provide qualitative feedback via open text boxes to comment on evidence or experiences that informed their responses.

Brief documents explaining key NMHSPF concepts and definitions of relevance to the questionnaire were developed to assist understanding and interpretation of questions. The Round 1 questionnaire was piloted with other researchers not directly part of this Delphi study to assess comprehension and readability.

#### 2.3.3 Delphi Process

Each Delphi questionnaire was open for two months; two reminder emails were sent for each round to encourage participation. Between rounds 1 and 2, quantitative results (i.e., the proportion of each NMHSPF need group who would likely benefit from lifestyle interventions) were analysed to calculate measures of central tendency (median) and level of dispersion (range). Qualitative feedback was thematically analysed by one author and then discussed with the research team. Prominent themes relevant to the study aim were incorporated as key themes for further consultation in the second round of the Delphi study.

#### 2.3.4 Delphi Analysis

Round 2 results were analysed to determine whether consensus had been achieved. We used the following rules to define when consensus agreement was achieved (Thomas et al. 2014):

- Strong consensus: ≥ 80% of panel members either ‘agree’ or ‘strongly agree’
- Moderate consensus: ≥ 60%–79% of panel members either ‘agree’ or ‘strongly agree’
- No consensus: < 60% of panel members either ‘agree’ or ‘strongly agree’

Quantitative results were exported as Microsoft Excel CSV files and analysed using Stata 16.

## 3 Results

### 3.1 Respondent Demographics

There were 25 unique participants across the Panel and Delphi. Fourteen participants completed the Round 1 Delphi questionnaire and seven completed Round 2. Across both the Panel and Delphi, more than half of the participants were clinicians (i.e., clinical academics or mental health service providers) (see Data S2). Lived experience representation was only achieved in the Panel. Four Australian states/territories were represented in the Panel (NSW = 5; Vic = 3; Qld = 3; WA = 1). Round 1 Delphi participants represented five jurisdictions (NSW = 9, Vic = 2, SA = 1, Qld = 1, WA = 1); Round 2 participants represented two (NSW = 6; Qld = 1).

### 3.2 Qualitative Results

During face‐to‐face meetings, the Panel discussed a range of topics including: the purpose and scope of lifestyle interventions; the benefits of the services; and operational factors to consider when planning and delivering these services. Table 1 summarises key discussion points.

**TABLE 1:** Key discussion points from the Panel meetings.

Definition	Lifestyle interventions are multidisciplinary services that aim to prevent and/or manage symptoms of mental disorders, comorbid conditions and the physical health side effects associated with certain psychotropic medications. They encompass educational and treatment components that aim to address health risk behaviours such as sedentary lifestyles, nutrition, tobacco use, alcohol and drug use, sleep hygiene and sexual health behaviours.
Benefits	Lifestyle interventions can help reduce or manage stress, improve symptoms of mental disorders and prevent disorder progression. Lifestyle interventions can help young adults develop the knowledge and skills required to develop and maintain healthy lifestyles, potentially having a positive lifelong impact on health. The use of qualified workforce (such as tertiary qualified dieticians and exercise physiologists, and peer workers) who have experience with or have undertaken additional training to deliver tailored support to people with mental health concerns. Group‐based services may provide additional benefits such as social skill development and improved social connectedness through peer interactions. The integration of lifestyle interventions into routine mental health care can increase engagement with other mental health services. Healthy lifestyle education is applicable to individuals of all severity levels and need groups.
Operational	Setting Service can be effectively delivered in either group‐based (e.g., six consumers and one practitioner leading an exercise or nutrition session) or individual settings (e.g., one‐on‐one consultations between the consumer and practitioner) depending on the needs and preferences of the individual. ○ Group‐based services may be more suited to individuals with higher cognitive and social functioning ○ Individual services allow clinical staff to provide one‐on‐one support including prescription of tailored diet and physical activity programs which may be more suitable for those with severe mental illness who may have lower cognitive and social functioning. Group‐based services may also incorporate individualised treatment methods however the clinician typically spends less one‐on‐one time with the individual. Duration An average of 6 sessions for the milder severity need groups (indicated prevention—low impairment, indicated prevention—high impairment, mild and moderate) and 12 sessions for the severe groups (severe—standard and severe—complex) was recommended Workforce A multidisciplinary team of clinical and non‐clinical staff (e.g., exercise physiologists, dieticians, physiotherapists, psychologists, nurses and peer workers) work together to provide a holistic service response, including but not limited to ○ Individual dietetic consultations and group‐based education delivered by dietitians on topics such as weight management, understanding nutrition labels, preparation of nutritious meals and food insecurity ○ Individual and group‐based exercise programs aiming to increase physical activity delivered by exercise physiologists ○ Support from psychologists and others to address alcohol and other drug problems, tobacco cessation and sleep hygiene ○ Health coaching to provide a motivational framework to assist with adherence to healthy lifestyles delivered by clinical nurse consultants ○ Peer workers are important members of the multidisciplinary teams; they participate in services such as cooking classes and exercise sessions, act as positive role models and provide motivation and encouragement for participants to remain engaged. The teams would ideally be integrated within existing mental health services (e.g., primary care and specialist community mental health teams) and have appropriate qualification or training in working with young adults with mental illness

Two key themes regarding lifestyle interventions were identified during Round 1 and presented to participants in Round 2. The first theme was ‘individuals in the severe – standard and severe – complex need groups would likely find it difficult to participate in group‐based interventions and therefore a larger proportion would benefit from individual services until symptoms are better managed’. Strong consensus agreement on this theme was achieved in Round 2 (71% ‘agree’; 14% ‘strongly agree’).

The second theme was ‘group‐based interventions are the gold standard of lifestyle interventions and should be the eventual goal of treatment as they provide the additional benefit of social support compared to individual lifestyle interventions’. There was a strong consensus disagreement found for this theme in Round 2 (43% ‘disagree’; 42% ‘strongly disagree’). Respondents indicated that gold standard practice should be a combination of both individual and group‐based lifestyle interventions with the former better able to provide personalised support and the latter providing social support.

### 3.3 Quantitative Results

The Panel deemed that lifestyle interventions were not required for the indicated prevention—low impairment need group but would play an important role for a small proportion of individuals in the indicated prevention—high impairment group. It was agreed that 30% of all other need groups (i.e., mild, moderate, severe—standard and severe—complex) would benefit from and be willing to access individually delivered interventions and 20% from group‐based interventions. The complete set of quantitative results recommended by the Panel is presented in Table 2.

**TABLE 2:** Proportion of each NMHSPF need group for young adults who would benefit from lifestyle interventions: Panel results versus Delphi results (%).

NMHSPF need groups	Panel results		Delphi results			
	Individually delivered	Group‐based	Individually delivered^a^	Consensus (% agree)	Group‐based^a^	Consensus (% agree)
Indicated Prevention—low impairment	n/a	n/a	80 (30–100)	100	70 (20–100)	86
Indicated Prevention—high impairment	15%	25%	90 (40–100)	71	70 (20–100)	71
Mild	30%	20%	80 (10–100)	86	70 (40–100)	100
Moderate	30%	20%	80 (30–100)	100	70 (50–100)	100
Severe—Standard	30%	20%	85 (30–100)	100	70 (40–100)	86
Severe—Complex	30%	20%	95 (50–100)	86	65 (30–100)	100

^a^ These columns represent the median score (i.e., the median proportion of young adults in each need group who may benefit from either individual or group‐based lifestyle interventions) as per the Round 1 Delphi results. These median scores were then presented in Round 2 of the Delphi to see if consensus could be achieved (i.e., that these scores represent a good estimate of the proportion of young adults who may benefit from lifestyle interventions).

Overall, Delphi respondents agreed that a high proportion of each need group would benefit from lifestyle interventions (Table 2). For indicated prevention need groups, respondents agreed that more people in the indicated prevention—high impairment group (90%) would benefit from individually delivered sessions compared to the indicated prevention—low impairment group (80%). For those with a diagnosed mental illness (i.e., mild, moderate, severe—standard, and severe—complex) the proportion who may benefit from individually delivered interventions increased with severity. For group‐based interventions, there was agreement that around 70% of all need groups would benefit; however, a slightly lower proportion was agreed upon for the severe‐complex need group (65%).

## 4 Discussion

### 4.1 Overall Findings

This study has drawn on the opinions of experts in youth mental health (including young people, carers and professionals working in the field) to generate evidence to inform population‐ and health‐service level planning for lifestyle interventions for young adults in Australia. This includes the identification of operational considerations for service commissioners and providers who may be designing and delivering lifestyle intervention services for young adults. The study has also developed provisional population‐level estimates of the proportion of young adults who are likely to benefit from lifestyle interventions as part of their mental health care. These can be used at population level, service level, or by individual clinicians to consider likely client needs for their populations and inform planning and delivery of lifestyle interventions to different client groups.

Experts agreed that there are a range of benefits to including lifestyle interventions as a core component of services that should be available to young adults experiencing mental health concerns. It was suggested that lifestyle interventions can be used to treat and improve symptoms of mental illness and promote healthy lifestyles which have positive mental and physical health impacts at present and into the future. Operational considerations for the design and planning of lifestyle interventions have also been outlined. It was recommended that interventions should be delivered in both individual and group‐based formats and it is important that consumer choice and preferences are catered to where possible. The study found that the duration of contact (e.g., number of sessions) to provide adequate care likely varies with severity, with greater numbers of sessions being recommended with increasing severity. Finally, it was emphasised that a mix of workforce types is required to work collaboratively as multidisciplinary teams to provide the necessary holistic care to young adults.

Experts in both the Panel and Delphi agree that lifestyle interventions should be available for young adults across the severity spectrum. This finding is consistent with the existing literature that has shown lifestyle interventions improved symptoms and functioning among a range of populations (Bailey et al. 2018; Carter et al. 2016; Curtis et al. 2018; Pascoe et al. 2020; Watkins et al. 2020). A higher proportion of people were estimated to need individual rather than group‐based services by both approaches used in this study. This contrasts current service provision in Australia, where lifestyle interventions tend to be delivered via group formats for people with mental disorders. This may be due to lower service delivery costs or a lack of understanding of the value and benefits of individual as well as group‐based formats.

This study has demonstrated that although consensus was achieved within each approach, there was a high level of discordance between approaches (Table 2) reflecting a lack of certainty over the proportion of young adults who would benefit from the services. The Delphi method produced significantly higher proportions across all need groups (e.g., 80%–95% from the Delphi method and around 30% for all need groups by the Panel). These differences could potentially be attributed to a range of factors.

First, the professional and lived experiences between the two groups were different. The Panel was comprised of youth mental health experts whose collective experience and expertise covered the spectrum of the mental health service system. Some Panel members held world leading expertise in lifestyle interventions, while others did not. However, individuals participating in the Delphi method had been specifically recruited based on their expertise in the benefits of lifestyle interventions.

Second, it is possible that the understanding of the purpose of the study (and reason for estimating proportions) varied between the two groups. Panel members received background information on the NMHSPF (written materials and a presentation at the first meeting) and were aware that the purpose of the Panel was to generate information for NMHSPF young adult care profiles. They had access to the complete care profiles and could review other services included to support mental health care.

Concerted efforts were made to ensure that Delphi participants were aware of the purpose of the study and were suitably grounded in NMHSPF concepts. This included provision of an example care profile to show how a range of other services could be received by young adults in each need group. However, it is possible that key messages and NMHSPF concepts may not have been as clearly understood, leading to higher proportions being recommended. For example, it is likely that estimates of the proportion who may need or benefit from lifestyle interventions would differ depending on whether the participants thought that was all they received to help manage symptoms of mental disorders or whether lifestyle interventions were one component of a broader suite of services received.

A third potential reason for the differences found between the two approaches could be the phrasing of Delphi questions and how the Panel was instructed to estimate proportions of young adults. When planning the Delphi method, a decision was made among the research team to phrase questions as “what proportion of each need group would *benefit* from lifestyle interventions.” However, during the Panel discussions a greater focus was on “what proportion of each need group would *need* lifestyle interventions.”

Finally, a strength of a Delphi method is its anonymity and that all participants can freely and independently voice their opinions without being influenced by dominant voices in a room. However, face‐to‐face Panel meeting discussions allowed rich discussion and debate among members. This, coupled with access to the complete care profile contents and a focus on ‘need’ over ‘benefit’, likely led to a natural moderation of the estimates for each service type within the care profiles.

### 4.2 Limitations

A motivation for conducting the Delphi method after the panel was to generate a broader consensus view (i.e., to involve a larger group of people) on the population need for lifestyle interventions. However, fewer people participated in the Delphi than the panel. If the Delphi approach was to be used in the future, more engaging communication methods, for example short videos or animations on the purpose of the study and value of people's contributions, should be considered to increase participation in online questionnaires. Additionally, peer review groups could be used or mental health service directors could be briefed on the study and invited to act as sponsors to encourage greater participation.

There was also no consumer or carer representation among Delphi survey respondents (despite invitations being distributed to peak bodies) which is of particular concern. The overarching aim of the NMHSPF care profiles is to reflect the average mix of services that may be needed over a 12‐month period to inform population‐based planning. It is essential that this vision of service need is informed by those who will be accessing services. It is possible that consumers and carers may prefer to voice opinions about service need in more nuanced discussions rather than responding to an online questionnaire. Consumer and carer representatives were also offered a stipend for participation on the Panel; however, there was no financial incentive to take part in the Delphi.

### 4.3 Service Implications

Literature on lifestyle interventions and mental illness has largely focused on people with severe mental illness (such as psychosis). However, there is a growing body of evidence that people with common mental disorders (such as depression and anxiety) and less severe presentations should also be provided with lifestyle interventions (Bailey et al. 2018; Pascoe et al. 2020). This study has added to this evidence by showing experts support lifestyle interventions as part of treatment across the full spectrum of diagnostic need groups, and as early intervention for people with sub‐threshold symptoms of mental illness (indicated prevention need groups).

Lifestyle intervention services are typically available in the specialist public mental health system for people with severe mental illness suffering from metabolic complications of their conditions and psychotropic medications. However, a recent Lancet Psychiatry Commission recommended that these services should be available in primary care potentially through ‘exercise referral schemes’ where clinician refer consumers to community‐based exercise programs at no or low cost to the consumer. This study provides additional support for more wide‐spread expansion of lifestyle intervention services across primary care and considering partnerships with non‐government organisations (Dray et al. 2022; Lederman et al. 2017; Opie et al. 2021).

Experts recommended that lifestyle interventions be delivered by multidisciplinary teams with appropriate qualifications or training to work with young adults with mental illness. This includes mental health nurses, exercise physiologists, dietitians, physiotherapists, peer workers, and psychologists. Nurses are well‐positioned to lead multidisciplinary teams and contribute to improved physical health and functional outcomes for those with mental illness due to their clinical skills and prominence in the mental health workforce (Fernández Guijarro et al. 2019; Happell et al. 2023). The upskilling of allied health professionals to provide discipline‐specific and holistic care to people with mental illness is particularly important given the shortage of traditional mental health providers (e.g., psychologists) within Australia (Australian Government Productivity Commission 2020). It was also suggested that lifestyle intervention services should be embedded within existing mental health services (e.g., primary care and specialist mental health teams), and that this integration can have an additional benefit of increasing engagement with other mental health care (Fehily et al. 2020; Lederman et al. 2017).

## 5 Conclusion

Experts agree that lifestyle interventions are beneficial and an important service component in the care of young adults with symptoms of or diagnosed mental illness. These services have a range of benefits for mental and physical health during young adulthood and potentially for improving health throughout the life course. This study sought to estimate the proportion of young adults who would benefit from lifestyle intervention services to provide pragmatic guidance to service planners when allocating resources and for clinicians designing care programmes for different client groups and in partnership with multidisciplinary teams. Despite the two approaches (discussions with a panel of experts and the Delphi method) independently achieving consensus on estimates, the discordance between the approaches demonstrates that the proportion of young people for whom lifestyle interventions need to be planned remains unclear.

Further research is needed to quantify the level of need among young adult populations and the likely uptake of these services so that all who may benefit and want to access these interventions as part of their mental health care can do so. Studies are also needed to confirm operational aspects of these interventions, including the efficient mix of workforce types and whether integration into typical mental health services (e.g., in primary care and specialist mental health teams) is the most efficient way of delivering care.

Overall, this study provides useful information for service planners and commissioners to understand some key inputs into the design of services, including the types of professionals needed, the intensity of service delivery required for different populations (e.g., indicated prevention, mild to severe presentations of mental illness) and the need to incorporate individual and group‐based service options. The use of this information in service planning will lead to increased availability of lifestyle interventions for young adults, yielding improved mental health, functioning, and physical health, and will support improved life outcomes.

## 6 Relevance for Clinical Practice

Lifestyle interventions are multidisciplinary services that aim to prevent and/or manage symptoms of mental disorders, functional impairment, comorbid conditions, and the physical health side effects associated with certain psychotropic medications. They encompass educational and treatment components addressing health risk behaviours such as sedentary lifestyles, nutrition, tobacco use, alcohol and drug use, sleep hygiene, and sexual health behaviours. Young people with all severities of mental illness are likely to benefit from lifestyle interventions as part of their care. Due to their educational background, nurses are uniquely placed within the mental health workforce to identify the need for lifestyle interventions among consumers, coordinate access to services, and deliver interventions. The integration of lifestyle interventions into routine mental health care can help to increase engagement with other mental health services. Interventions should be offered in both individual and group formats and integrated within existing primary care and specialist mental health services.

## Author Contributions

K.G., J.J., C.C., and S.D. conceived the study, prepared research materials, facilitated original expert panel meetings, and designed and provided input into the Delphi process. K.G. conducted the analysis, and I.P. helped with the analysis and write‐up of data. K.G. wrote the first draft of the manuscript, and C.C. revised it. H.E.E., J.G.S., and S.D. provided ongoing discussion and feedback on drafts. All authors approved the final version.

## Ethics Statement

This study was approved by the Human Research Ethics Committee of The University of Queensland (Ethics IDs 2020001887 and 2020001878).

## Conflicts of Interest

The authors declare no conflicts of interest.

## Supporting information


Data S1.

Data S2.

## Data Availability

Data collected and analysed for this study are not able to be shared due to ethics and confidentiality agreements.

## References

[inm70034-bib-0001] Australian Government Productivity Commission . 2020. “Productivity Commission Inquiry Report: Mental Health.”

[inm70034-bib-0002] Bailey, A. , S. Hetrick , S. Rosenbaum , R. Purcell , and A. Parker . 2018. “Treating Depression With Physical Activity in Adolescents and Young Adults: A Systematic Review and Meta‐Analysis of Randomised Controlled Trials.” Psychological Medicine 48, no. 7: 1068–1083. 10.1017/S0033291717002653. 28994355

[inm70034-bib-0003] Bailey, J. M. , K. Bartlem , J. H. Wiggers , et al. 2019. “Systematic Review and Meta‐Analysis of the Provision of Preventive Care for Modifiable Chronic Disease Risk Behaviours by Mental Health Services.” Preventive Medicine Reports 16: 100969. 31497500 10.1016/j.pmedr.2019.100969 PMC6718945

[inm70034-bib-0004] Bartlem, K. , J. A. Bowman , M. Freund , et al. 2015. “Acceptability and Receipt of Preventive Care for Chronic‐Disease Health Risk Behaviors Reported by Clients of Community Mental Health Services.” Psychiatric Services 66, no. 8: 857–864. 10.1176/appi.ps.201400360. 25930044

[inm70034-bib-0005] Carney, R. , S. Imran , H. Law , J. Firth , and S. Parker . 2021. “Physical Health Interventions on Adolescent Mental Health Inpatient Units: A Systematic Review and Call to Action.” Early Intervention in Psychiatry 15, no. 3: 439–448. 10.1111/eip.12981. 32426944

[inm70034-bib-0006] Carter, T. , I. Morres , J. Repper , and P. Callaghan . 2016. “Exercise for Adolescents With Depression: Valued Aspects and Perceived Change.” Journal of Psychiatric and Mental Health Nursing 23: 37–44. 26289604 10.1111/jpm.12261

[inm70034-bib-0007] Çelik İnce, S. , and N. Partlak Günüşen . 2021. “Effect of a Nurse‐Led Intervention Program on the Physical Health and Quality of Life of Individuals With Severe Mental Illness.” Perspectives in Psychiatric Care 57: 1751–1764. 10.1111/ppc.12745. 33616211

[inm70034-bib-0008] Colizzi, M. , A. Lasalvia , and M. Ruggeri . 2020. “Prevention and Early Intervention in Youth Mental Health: Is It Time for a Multidisciplinary and Trans‐Diagnostic Model for Care?” International Journal of Mental Health Systems 14, no. 23: 23. 10.1186/s13033-020-00356-9. 32226481 PMC7092613

[inm70034-bib-0009] Cox, G. R. , C. A. Fisher , S. De Silva , et al. 2012. “Interventions for Preventing Relapse and Recurrence of a Depressive Disorder in Children and Adolescents.” Cochrane Database of Systematic Reviews 11, no. 11: CD007504. 10.1002/14651858.cd007504.pub2. 23152246 PMC8978530

[inm70034-bib-0010] Curry, J. , S. Silva , P. Rohde , et al. 2011. “Recovery and Recurrence Following Treatment for Adolescent Major Depression.” Archives of General Psychiatry 68, no. 3: 263–269. 10.1001/archgenpsychiatry.2010.150. 21041606 PMC3074362

[inm70034-bib-0011] Curtis, J. , A. Watkins , S. Rosenbaum , et al. 2016. “Evaluating an Individualized Lifestyle and Life Skills Intervention to Prevent Antipsychotic‐Induced Weight Gain in First‐Episode Psychosis.” Early Intervention in Psychiatry 10, no. 3: 267–276. 10.1111/eip.12230. 25721464

[inm70034-bib-0012] Curtis, J. , A. Watkins , S. Teasdale , et al. 2018. “2‐Year Follow‐Up: Still Keeping the Body in Mind.” Australian and New Zealand Journal of Psychiatry 52, no. 6: 602–603. 10.1177/0004867417753553. 29359572

[inm70034-bib-0013] Di Rezze, B. , T. Nguyen , G. Mulvale , N. G. Barr , C. Longo , and G. Randall . 2016. “A Scoping Review of Evaluated Interventions Addressing Developmental Transitions for Youth With Mental Health Disorders.” Child: Care, Health and Development 42, no. 2: 176–187. 10.1111/cch.12306. 26638809

[inm70034-bib-0014] Diminic, S. , K. Gossip , I. Page , and C. Comben . 2022. “Introduction to the National Mental Health Service Planning Framework – Commissioned by the Australian Government Department of Health.” Version AUS V4.2. The University of Queensland, Brisbane. https://www.aihw.gov.au/nmhspf/overview/documentation.

[inm70034-bib-0015] Dray, J. , L. Gibson , T. Clinton‐McHarg , et al. 2022. “Exploring Support Provided by Community Managed Organisations to Address Health Risk Behaviours Associated With Chronic Disease Among People With Mental Health Conditions: A Qualitative Study With Organisational Leaders.” International Journal of Environmental Research and Public Health 19, no. 9: 5533. 10.3390/ijerph19095533. 35564928 PMC9105164

[inm70034-bib-0016] Fehily, C. , K. Bartlem , J. H. Wiggers , et al. 2020. “Effectiveness of Embedding a Specialist Preventive Care Clinician in a Community Mental Health Service in Increasing Preventive Care Provision: A Randomised Controlled Trial.” Australian and New Zealand Journal of Psychiatry 54, no. 6: 620–632. 32403938 10.1177/0004867420914741 PMC7285986

[inm70034-bib-0017] Fernández Guijarro, S. , E. Pomarol‐Clotet , M. C. Rubio Muñoz , et al. 2019. “Effectiveness of a Community‐Based Nurse‐Led Lifestyle‐Modification Intervention for People With Serious Mental Illness and Metabolic Syndrome.” International Journal of Mental Health Nursing 28, no. 6: 1328–1337. 10.1111/inm.12644. 31411375

[inm70034-bib-0018] Gibb, S. J. , D. M. Fergusson , and L. J. Horwood . 2010. “Burden of Psychiatric Disorder in Young Adulthood and Life Outcomes at Age 30.” British Journal of Psychiatry 197, no. 2: 122–127. 10.1192/bjp.bp.109.076570. 20679264

[inm70034-bib-0019] Gossip, K. , J. John , C. Comben , et al. 2022. “Key Service Components for Age‐Appropriate Mental Health Service Planning for Young Adults.” Early Intervention in Psychiatry 16, no. 10: 1085–1093. 10.1111/eip.13253. 34821037

[inm70034-bib-0020] Happell, B. , T. Furness , A. Jacob , et al. 2023. “Nurse‐Led Physical Health Interventions for People With Mental Illness: A Scoping Review of International Literature.” Issues in Mental Health Nursing 44, no. 6: 458–473. 10.1080/01612840.2023.2212772. 37294933

[inm70034-bib-0021] Happell, B. , C. Platania‐Phung , A. Watkins , et al. 2019. “Developing an Evidence‐Based Specialist Nursing Role to Improve the Physical Health Care of People With Mental Illness.” Issues in Mental Health Nursing 40, no. 10: 832–838. 10.1080/01612840.2019.1584655. 31070501

[inm70034-bib-0022] IHME . 2019. “GBD Compare.” University of Washington. https://vizhub.healthdata.org/gbd‐compare/.

[inm70034-bib-0023] Iorfino, F. , J. S. Carpenter , S. P. Cross , et al. 2022. “Social and Occupational Outcomes for Young People Who Attend Early Intervention Mental Health Services: A Longitudinal Study.” Medical Journal of Australia 216, no. 2: 87–93. 10.5694/mja2.51308. 34664282

[inm70034-bib-0024] Iorfino, F. , D. Hermens , S. Cross , et al. 2018. “Delineating the Trajectories of Social and Occupational Functioning of Young People Attending Early Intervention Mental Health Services in Australia: A Longitudinal Study.” BMJ Open 8, no. 3: e020678. 10.1136/bmjopen-2017-020678. PMC5875606 29588325

[inm70034-bib-0025] Jones, P. B. 2013. “Adult Mental Health Disorders and Their Age at Onset.” British Journal of Psychiatry 202, no. 54: s5–s10. 10.1192/bjp.bp.112.119164. 23288502

[inm70034-bib-0026] Jorm, A. 2015. “Using the Delphi Expert Consensus Method in Mental Health Research.” Australian and New Zealand Journal of Psychiatry 49, no. 10: 887–897. 26296368 10.1177/0004867415600891

[inm70034-bib-0027] Knapp, M. , V. Ardino , N. Brimblecombe , et al. 2016. “Youth Mental Health: New Economic Evidence.”

[inm70034-bib-0028] Lederman, O. , S. Suetani , R. Stanton , et al. 2017. “Embedding Exercise Interventions as Routine Mental Health Care: Implementation Strategies in Residential, Inpatient and Community Settings.” Australasian Psychiatry 25: 451–455. 28585448 10.1177/1039856217711054

[inm70034-bib-0029] Malhi, G. S. , D. Bassett , P. Boyce , et al. 2015. “Royal Australian and New Zealand College of Psychiatrists Clinical Practice Guidelines for Mood Disorder.” Australian and New Zealand Journal of Psychiatry 49, no. 12: 1087–1206. 10.1177/0004867415617657. 26643054

[inm70034-bib-0030] Manager, S. 2019. “Lifestyle Interventions for Mental Health.” Australian Journal of General Practice 48, no. 10: 670–673. 10.31128/AJGP-06-19-4964. 31569326

[inm70034-bib-0031] O'Cathain, A. , E. Murphy , and J. Nicholl . 2017. “The Quality of Mixed Methods Studies in Health Services Research.” Journal of Health Services Research & Policy 13, no. 2: 92–98. 10.1258/jhsrp.2007.007074 18416914

[inm70034-bib-0032] Opie, R. S. , F. N. Jacka , M. Marx , T. Rocks , C. Young , and A. O'Neil . 2021. “Designing Lifestyle Interventions for Common Mental Disorders: What Can we Learn From Diabetes Prevention Programs?” Nutrients 13, no. 11: 3766. 10.3390/nu13113766. 34836024 PMC8619252

[inm70034-bib-0033] Pascoe, M. , A. P. Bailey , M. Craike , et al. 2020. “Physical Activity and Exercise in Youth Mental Health Promotion: A Scoping Review.” BMJ Open Sport & Exercise Medicine 6, no. 1: e000677. 10.1136/bmjsem-2019-000677. PMC7010991 32095272

[inm70034-bib-0034] Patel, V. , A. Flisher , S. Hetrick , and P. McGorry . 2007. “Mental Health of Young People: A Global Public‐Health Challenge.” Lancet 369, no. 9569: 1302–1313. 10.1016/s0140-6736(07)60368-7. 17434406

[inm70034-bib-0035] Stubbs, B. , D. Vancampfort , M. Hallgren , et al. 2018. “EPA Guidance on Physical Activity as a Treatment for Severe Mental Illness: A Meta‐Review of the Evidence and Position Statement From the European Psychiatric Association.” European Psychiatry 54: 124–144. 30257806 10.1016/j.eurpsy.2018.07.004

[inm70034-bib-0036] Thomas, S. , J. Wakerman , and J. Humphreys . 2014. “What Core Primary Health Care Services Should Be Available to Australians Living in Rural and Remote Communities?” BMC Family Practice 15, no. 1: 143. 10.1186/1471-2296-15-143. 25143194 PMC4236500

[inm70034-bib-0037] Vos, T. , R. M. Barber , B. Bell , et al. 2015. “Global, Regional, and National Incidence, Prevalence, and Years Lived With Disability for 301 Acute and Chronic Diseases and Injuries in 188 Countries, 1990–2013: A Systematic Analysis for the Global Burden of Disease Study 2013.” Lancet 386, no. 9995: 743–800. 10.1016/s0140-6736(15)60692-4. 26063472 PMC4561509

[inm70034-bib-0038] Watkins, A. , E. Denney‐Wilson , J. Curtis , et al. 2020. “Keeping the Body in Mind: A Qualitative Analysis of the Experiences of People Experiencing First‐Episode Psychosis Participating in a Lifestyle Intervention Programme.” International Journal of Mental Health Nursing 29, no. 2: 278–289. 10.1111/inm.12683. 31840386

