# Supplementary material for: Estimating Population‐Based Need for Lifestyle Interventions Among Young Adults With Mental Disorders in Australia
Source: Int J Ment Health Nurs. 2025 Apr 4;34(2):e70034. doi: 10.1111/inm.70034 (PMC11969636; doi:10.1111/inm.70034)
Supplement: Supplementary file 1 — Data S1. [file INM-34-0-s001.pdf]

Supplementary Appendix 1. Lifestyle interventions Delphi survey questionnaire

1. Lifestyle interventions – introduction

Background

Lifestyle interventions seek to improve both mental health and physical health in people with mental illness. They are multidisciplinary interventions that may focus on physical activity and exercise, nutrition, sleep hygiene, alcohol and drug use, sexual health and smoking cessation. These interventions can be delivered one-one-one or in group-based settings by qualified professionals. Lifestyle interventions have been shown to produce positive outcomes for individuals who experience different types of mental illnesses and at different levels of illness severity across the lifespan[1].

While there is strong evidence to support the use of lifestyle interventions as a treatment for people with mental illness, such interventions are not widely available within the Australian mental health system. To assist policy makers and service planners, existing research evidence needs to be translated into pragmatic service planning guidance. More specifically, from a needs-based planning perspective, an indication of how many people with mental illness are likely to need these interventions is required.

This study will use the Delphi method to generate consensus on the proportion of people aged 18-24 years who are likely to benefit from lifestyle interventions. The results of the study will be used to inform modelling in the Australian National Mental Health Service Planning Framework (NMHSPF), a tool used to guide service planning.

The questions in this Delphi survey will ask you to think about your personal and professional experiences to estimate a proportion of each NMHSPF need group, who are likely to benefit from individually delivered or group-based lifestyle interventions.

[1] Firth et al, A meta-review of "lifestyle psychiatry": the role of exercise, smoking, diet and sleep in the prevention and treatment of mental disorders. World Psychiatry 2020;19:360-380.

2. Young adult survey questions

The following questions will ask you what proportion of each of the following NMHSPF need groups for young adults (18-24 years) would benefit from having access to individually delivered or group-based lifestyle interventions. Please answer this based on your experiences (i.e. clinical case load, service user perspectives). Please answer the questions to the best of your knowledge, however you may select 'Unsure' if you are uncertain in your opinion. **This information sheet includes all the definitions and detail about the young adults (18-24 years) age group.**

What proportion of young adults (18-24 years) in the indicated prevention – low impairment need group would benefit from individually delivered lifestyle interventions?

Unsure 0% 10% 20% 30% 40% 50% 60% 70% 80% 90% 100%

What proportion of young adults (18–24 years) in the indicated prevention – low impairment need group would benefit from group-based lifestyle interventions?

Unsure 0% 10% 20% 30% 40% 50% 60% 70% 80% 90% 100%

What proportion of young adults (18–24 years) in the indicated prevention – high impairment need group would benefit from individually delivered lifestyle interventions?

Unsure 0% 10% 20% 30% 40% 50% 60% 70% 80% 90% 100%

What proportion of young adults (18–24 years) in the indicated prevention – high impairment need group would benefit from group-based lifestyle interventions?

Unsure 0% 10% 20% 30% 40% 50% 60% 70% 80% 90% 100%

What proportion of young adults (18–24 years) in the mild need group would benefit from individually delivered lifestyle interventions?

Unsure 0% 10% 20% 30% 40% 50% 60% 70% 80% 90% 100%

What proportion of young adults (18–24 years) in the mild need group would benefit from group-based lifestyle interventions?

Unsure 0% 10% 20% 30% 40% 50% 60% 70% 80% 90% 100%

What proportion of young adults (18–24 years) in the moderate need group would benefit from individually delivered lifestyle interventions?

Unsure 0% 10% 20% 30% 40% 50% 60% 70% 80% 90% 100%

What proportion of young adults (18–24 years) in the moderate need group would benefit from group-based lifestyle interventions?

Unsure 0% 10% 20% 30% 40% 50% 60% 70% 80% 90% 100%

What proportion of young adults (18–24 years) in the severe - standard need group would benefit from individually delivered lifestyle interventions?

Unsure 0% 10% 20% 30% 40% 50% 60% 70% 80% 90% 100%

What proportion of young adults (18–24 years) in the severe - standard need group would benefit from group-based lifestyle interventions?

Unsure 0% 10% 20% 30% 40% 50% 60% 70% 80% 90% 100%

What proportion of young adults (18–24 years) in the severe - complex need group would benefit from individually delivered lifestyle interventions?

Unsure 0% 10% 20% 30% 40% 50% 60% 70% 80% 90% 100%

What proportion of young adults (18–24 years) in the severe - complex need group would benefit from group-based lifestyle interventions?

Unsure 0% 10% 20% 30% 40% 50% 60% 70% 80% 90% 100%

If you have any further comments or suggestions in response to the questions above, feel free to provide in the comment box below.

Comment box

NMHSPF Young Adults (18-24 years) age group

This document includes all the NMHSPF definitions and details relevant to the young adult age group.

Lifestyle interventions

- For the purposes of this study, lifestyle interventions are defined as multidisciplinary interventions that aim to improve and/or manage symptoms of mental illness, comorbid physical health conditions and side effects of medication. Interventions may focus on physical activity and exercise, nutrition, sleep hygiene, alcohol and drug use, sexual health and smoking cessation.
- These interventions are delivered by suitably qualified professionals with experience working with populations with mental illness. Professions may include exercise physiologists, dieticians, peer workers and clinical nurse consultants.
- Lifestyle interventions may be delivered in a one-on-one setting between the consumer and professional, or in a group-based setting.

NMHSPF severity levels

- In the NMHSPF, the severity levels (i.e., *Indicated Prevention, Relapse Prevention, Mild, Moderate and Severe*) have very specific meanings. The severity levels refer to the intensity of mental health service needs for people with symptoms or formally diagnosed mental illness, which is more closely related to role impacts and impairment in psychosocial functioning than types of clinical symptoms.

NMHSPF need groups

- The need groups represent a group of people who experience a similar level of impairment and complexity associated with mental illness. Individuals within the group, on average, require similar types of services although individual needs may vary from person to person. The mix of service types and level of care required (in terms of the types of services needed, frequency and duration of contact with services) varies across need groups with severity.

Table 1 below provides definitions for each of the need groups within the severity levels that are relevant for this questionnaire.

Table 2 demonstrates how the types of services needed by people in each need group changes with severity.

Table A1. Need group definitions for the 18-24 years age group

Severity levels and need groups	Definition
**Indicated prevention**	
**Indicated prevention – low impairment**	This need group includes individuals who are experiencing symptoms of mental illness but do not meet the threshold for a formal diagnosis. While their symptoms are currently having a 'low' impact on their day to day functioning (e.g. ability to participate in education or employment, or fulfill family responsibilities) they would benefit from interventions focused on coping strategies and preventing progression to a formal diagnosis.
**Indicated prevention – high impairment**	This need group includes individuals who are experiencing symptoms of mental illness but do not meet the threshold for a formal diagnosis. Their symptoms and other social and environmental factors are having a 'high impact' on their functioning. For example, they may not feel that they are able to participate fully in their education or employment, or may be having significant relationship problems with families and friends. Individuals in this need group would benefit from holistic interventions focused on addressing the symptoms and functional impairments currently being experienced to improve wellbeing and prevent progression to a formal diagnosis.
**Relapse prevention**	
**Relapse prevention**	The relapse prevention need group includes people who have previously had a formal diagnosis of mental illness. At present they do not currently meet criteria for mental illness but would benefit from access to supports to help them to remain well.
**Mild**	
**Mild**	This need group includes individuals who have a diagnosed mental illness that has a *low* impact on their day-to-day lives. This group of individuals require contact with mental health services and would benefit from holistic interventions to address symptoms and associated functional impairment.
**Moderate**	
**Moderate**	This need group includes individuals who have a diagnosed mental illness that has *moderate* impact on their day-to-day lives. They may experience problems in psychosocial functioning (e.g. in the workplace, education, household or social settings) resulting in reduced productivity, performance and engagement. Many of these individuals are likely to experience multiple mental and/or physical illnesses and may also have risk factors such as extensive social and environmental stressors. This group of individuals require regular contact with mental health services and would benefit from holistic and multidisciplinary interventions to address symptoms and associated functional impairment.
**Severe**	
**Severe – standard**	This need group includes individuals that have a diagnosed mental illness that causes *a high* impact on their day-to-day lives. They experience problems with their psychosocial functioning which can have substantial impacts on their usual roles in a range of settings (e.g. employment, education, household and social). Individuals in this group require a holistic and multidisciplinary service response that includes regular contact with services and input from specialist providers (e.g. psychiatrists).<br><br>The majority of individuals receive care via community-based services. However, a small proportion may experience increased risk (to themselves or others) and require short term care within bed-based services such as hospital based acute inpatient services and

	step up/step down services in residential settings to receive intensive treatment and manage risks.
**Severe – complex**	This need group includes individuals that have a diagnosed mental illness that causes *a high* impact on their day-to-day lives. They experience significant impairment in psychosocial functioning that causes problems in their ability to participate in education and employment, and engage in meaningful relationships and social activities. They are likely to have multiple mental and or physical health problems and many will also experience extensive social and environmental stressors that complicate their care needs. All individuals require an intensive holistic and multidisciplinary service response from specialist services (e.g. public sector community teams).<br><br>The majority of individuals receive care via community-based services. However, a small proportion may experience increased risk (to themselves or others) and require short term care within bed-based services such as hospital based acute inpatient services and step up/step down services in residential settings to receive intensive treatment and manage risks. Some may also require longer-term care in hospital or residential settings to receive recovery oriented care focused on building links with the community, sustaining social connectedness, developing skills to enable self-management of mental and general health care and supporting vocational goals.

Table A2. Types of services currently modelled to be received by each need group in the 18-24 years age group

	Primary care					Public sector teams		Structured psychological therapies (SPT)					Psychosocial support services					Bed-based services		
	Assessments	Review +/- ongoing management	Care coordinati-on and liaison	Pharmacothe-rapy prescription and review	#Lifestyle Interventions	Clinical community treatment team	Acute care services	Clinician moderated web-based SPT	Low intensity SPT	Brief SPT	Individual and/or group SPT	Family SPT	Individual and/or group-based consumer peer work	Individual and/or group-based carer peer work	Family support services	Vocational support services	Youth developme-nt services	Acute inpatient services (hospital settings)	Step up/step down services (residential settings)	Sub-acute service and non-acute services (hospital or residential settings)
**Indicated prevention – low impairment**																				
**Indicated prevention – high impairment**																				
**Relapse prevention**																				
**Mild**																				
**Moderate**																				
**Severe – standard**																				
**Severe – complex**																				

# The need for lifestyle interventions in the NMHSF need groups is the focus of this Delphi study.
