# Supplementary material for: Estimating Population‐Based Need for Lifestyle Interventions Among Young Adults With Mental Disorders in Australia
Source: Int J Ment Health Nurs. 2025 Apr 4;34(2):e70034. doi: 10.1111/inm.70034 (PMC11969636; doi:10.1111/inm.70034)
Supplement: Supplementary file 2 — Data S2. [file INM-34-0-s002.pdf]

Supplementary Appendix 2. NMHSPF Youth Expert Panel member backgrounds, meeting attendance and overall participant expertise

The 12-person Panel included seven managers of youth mental health services, most of whom were also clinicians (three psychiatrists, two psychologists and one mental health nurse), four young adults with lived experience of mental illness (one of whom was also a carer of a young adult with mental illness), and one carer who was a parent of a young adult with a mental illness. Collectively, the Panel had experience across all sectors of the Australian mental health system, including primary care, specialist public sector, and non-government organisations.

Table A3. Panel member backgrounds and meeting attendance

YEP member no.	Area of expertise	Jurisdiction	YEP meetings*				Out of session meetings
			YEP 1: 24-25/07/2019	YEP 2: 6-7/11/2019	YEP 3: 20-21/02/2020	YEP 4: 16-17/04/2020	First episode psychosis services: 15/01/2020
1	SM, A	ACT	✓	✓	✓	✓	
2	C, SM, A	NSW	✓	✓	✓	✓	✓
3	C, SM, A	Vic	✓	✓	✓	✓	✓
4	Ca	NSW	✓	✓		✓	
5	C, SM	Vic	✓		✓	✓	
6	C, SM	Qld	✓	✓	✓		
7	C, SM	Vic	✓	✓	✓	✓	
8	C, SM	NSW		✓	✓	✓	
9	Co	WA	✓				
10	Co	Vic		✓			
11	Co	Qld			✓		
12	Ca, Co	Qld			✓	✓	

Area of expertise: Academic/researcher (A), Carer (Ca), Clinician (C), Consumer (Co), Service Manager (SM).
Jurisdiction (state or territory in which the members reside): Australian Capital Territory (ACT), New South Wales (NSW), Queensland (Qld), Victoria (Vic), Western Australia (WA).
*Not all consumers were able to attend all meetings but we ensured that at least one young adult consumer was able to participate in each meeting.

There were also an additional 40 individuals from across Australia consulted on specific questions bilaterally between meetings as part of the development of the NMHSPF care profiles for young adults. This included clinicians, academics and service managers from Local Hospital Networks, Primary Health Networks, Jurisdictional and national health departments.

Expertise of Panel members and Delphi respondents

There was a total of 25 unique participants between the Panel and Delphi respondents. One participant was represented in both the Panel and the Delphi.

Table A4. Panel and Delphi respondent expertise

**Expertise**	**Panel members n (%)**	**Respondents to Round 1 Delphi questionnaire n (%)**	**Respondents to Round 2 Delphi questionnaire n (%)**
Clinical academic	3 (25%)	7 (50%)	2 (29%)
Academic	Nil	3 (21%)	3 (42%)
Mental health service provider	4 (33%)	4 (29%)	2 (29%)
Carer	1 (8%)	Nil	Nil
Consumer	4 (33%)	Nil	Nil
**Total participants**	**12**	**14**	**7**
